# Microbial Photoinactivation by Visible Light Results in Limited Loss of Membrane Integrity

**DOI:** 10.3390/antibiotics10030341

**Published:** 2021-03-23

**Authors:** Katharina Hoenes, Richard Bauer, Barbara Spellerberg, Martin Hessling

**Affiliations:** 1 Institute of Medical Engineering and Mechatronics, Ulm University of Applied Sciences, 89081 Ulm, Germany; martin.hessling@thu.de; 2 Institute of Medical Microbiology and Hygiene, University Hospital Ulm, 89081 Ulm, Germany; richard.bauer@uni-ulm.de (R.B.); Barbara.Spellerberg@uniklinik-ulm.de (B.S.)

**Keywords:** photoinactivation, antimicrobial blue light (aBL), reactive oxygen species (ROS), membrane damage, membrane permeabilization, 405 nm, 450 nm

## Abstract

Interest in visible light irradiation as a microbial inactivation method has widely increased due to multiple possible applications. Resistance development is considered unlikely, because of the multi-target mechanism, based on the induction of reactive oxygen species by wavelength specific photosensitizers. However, the affected targets are still not completely identified. We investigated membrane integrity with the fluorescence staining kit LIVE/DEAD^®^ BacLight™ on a Gram positive and a Gram negative bacterial species, irradiating *Staphylococcus carnosus* and *Pseudomonas fluorescens* with 405 nm and 450 nm. To exclude the generation of viable but nonculturable (VBNC) bacterial cells, we applied an ATP test, measuring the loss of vitality. Pronounced uptake of propidium iodide was only observed in *Pseudomonas fluorescens* at 405 nm. Transmission electron micrographs revealed no obvious differences between irradiated samples and controls, especially no indication of an increased bacterial cell lysis could be observed. Based on our results and previous literature, we suggest that visible light photoinactivation does not lead to rapid bacterial cell lysis or disruption. However, functional loss of membrane integrity due to depolarization or inactivation of membrane proteins may occur. Decomposition of the bacterial envelope following cell death might be responsible for observations of intracellular component leakage.

## 1. Introduction

Bacterial photoinactivation with visible light represents a promising microbial inactivation strategy, with many advantages and various potential applications [1,2,3,4,5,6,7]. Violet light with a wavelength of around 405 nm seems to be especially effective [8,9], but also blue light around 450 nm exhibits antimicrobial properties [10,11]. Those are based on endogenous photosensitizers absorbing light of specific wavelengths [4,12], inducing the formation of reactive oxygen species (ROS) [13,14], which then attack intracellular bacterial targets. 

Studying underlying conditions and optimal parameters, the mode of action was addressed, determining different inactivation mechanisms in multiple investigations. Among the cellular targets, the cell membrane was often named as an important site [15,16,17,18,19], but also other molecular targets have been recognized [20,21,22,23,24]. Oxidation of nucleic acids was often observed [21,22,24,25]. However, no breakage of the DNA strand could be detected [18,19], while genetic changes in the oxidative stress pathway were elucidated [25,26]. Membrane permeabilization was concluded mainly from observation of leaked intracellular material [15,16,27,28,29,30] and from penetration of stains in some studies [15,18,19,26,29]. A later study of one of those research groups did not reveal any staining, but loss of glucose uptake and efflux activity instead [21], whereas loss of potassium and phosphate, detected by other authors [4,31], similarly point towards inactivation of ion pumps.

Some of the studies contradict each other and it seems to be difficult to carve out a specific cause of death. The precise mechanism of action for photoinactivation with visible light has therefore not been fully elucidated. It is however not unlikely that several of those studies are equally valid, as the inactivation process is conducted by ROS, acting as mediators between light absorbing site and bacterial damage. There is a high probability of multiple targets being responsible for the reduction in bacterial viability. Each microorganism contains not only one, but several sites sensitive to oxidative stress and at the same time necessary to maintain vitality.

The decreased risk of resistance development against the irradiation with visible light noticed so far [27,32,33], is presumably due to this non-specific damage, so that specific efforts of the microorganism to mobilize bypassing strategies result in moderate consequences. Nevertheless, the possibility of resistance acquisition cannot be excluded in general [34,35].

Yet it is important to determine the main responsible factors for bacterial inactivation for a better comprehension of this inactivation technique leading to a more purposeful and effective action, which is also important for a transfer to realistic applications.

Addressing the question of the contribution of membrane damage to the inactivation success, we previously performed a study examining the impact of visible light of 405 and 450 nm on the yeast *Saccharomyces cerevisiae* [36]. We applied a trypan blue assay in parallel to colony forming unit (CFU) determination and found highly differing results. Trypan blue assay is a well-established procedure to distinguish between live and dead microorganisms based on the decreasing membrane integrity [37,38]. Despite the decrease of viable yeast by three log levels (99.9%) at a dose of 583.2 J/cm^2^ with 405 nm irradiation determined with CFU assessment, only approximately 30% of yeast cells exhibited membrane damage. Assuming, that membrane damage is an important cause of death in the mechanism of visible light irradiation, as it was mentioned in the literature before, and can therefore be used to identify photoinactivated microorganisms, the approach of microscopic assessment would only suggest an approximate 0.15 log level reduction in the yeast’s viability after a dose of 583.2 J/cm^2^. Compared to the three log levels determined by CFU assessment this leads to doubts about the relevance of the contribution of membrane permeabilization. 

In microbiological investigations, researchers have to rely on methods that indirectly provide information about the viability of a microorganism. Those methods can be classified in two different groups [39], the first one including methods being based on the growth of microorganisms on solid or in liquid medium, while the second bundle of approaches contains staining procedures. The widely applied determination of colony forming units belongs to the first group and likewise the determination of inhibition zones by an antimicrobial agent or growth curves in liquid medium. However, these methods fail to provide information about microorganisms that are still viable but have lost the ability to reproduce. For investigation of photoinactivation with visible light mostly growth-dependent methods have been applied [9,11,40,41,42,43,44]. The second group of methods including staining with colorimetric or fluorescent dyes are dependent on the membrane properties. Some dyes are excluded by the cell envelope and are only able to penetrate compromised membranes, others penetrate microorganisms in all states and are actively pumped out or reduced in other ways by living cells. Only the last-mentioned methods are based on the physiological state of the microorganism determining its vitality, while procedures measuring the viability do not take into account slightly damaged bacteria, unable to divide but still vital. Methods determining the vitality might be based on the measurement of metabolic or enzymatic activity or on the content of ATP [45].

The interpretation of the deviations of results obtained with different methods on *S. cerevisiae* [36] can therefore lead to different conclusions. Obviously, membrane damage plays a minor role for light induced inactivation, at least for *S. cerevisiae*. We hypothesize that this might also be true for bacteria, contradictory of earlier assumptions in the literature. Another possible interpretation suggests not only an intact membrane for most cells, but the possibility of not having reduced the viable number of microorganisms, but only their culturability.

Due to these considerations we aimed to perform the test of membrane integrity after visible light irradiation on bacteria in this study based on the comparison of CFU assessment and staining results. We complemented the investigation with an assay addressing physiological parameters, namely the measurement of ATP content, to exclude mere culturability loss at intact viability in case of a stable membrane. *Staphylococcus carnosus* and *Pseudomonas fluorescens* were investigated under 405 nm and 450 nm irradiation, representing a Gram positive and a Gram negative example, as results concerning membrane response might vary due to structural differences.

## 2. Results

### 2.1. Viability Determination with Colony Forming Units

**Figure 1:**
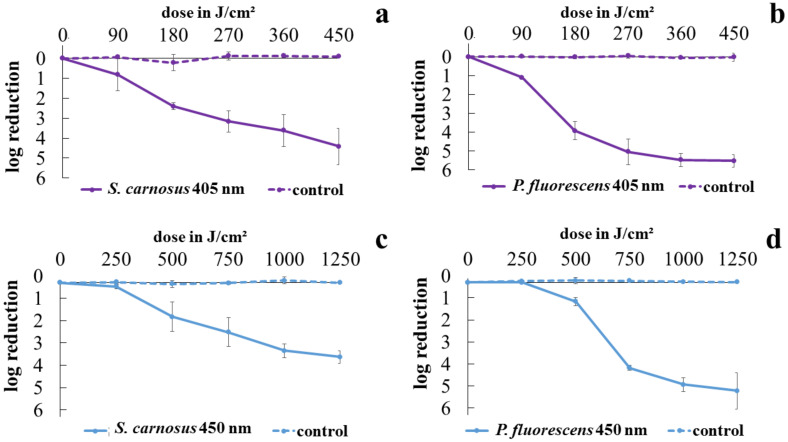
Inactivation progress of *S. carnosus* irradiated with 405 nm (**a**) and 450 nm (**c**) as well as inactivation progress of *P. fluorescens* at 405 nm (**b**) and 450 nm (**d**). Error bars are representing the standard deviation of three independent experiments.

The results of viability determination with CFU for *S. carnosus* and *P. fluorescens* irradiated with 405 nm or 450 nm are depicted in Figure 1. For all irradiated samples, the final dose was sufficient to reach a reduction of over three log levels. *P. fluorescens* was more susceptible to both investigated wavelengths compared to *S. carnosus*. For 405 nm irradiation at the final dose of 450 J/cm^2^, a reduction of 5.52 log levels was reached for pseudomonads, whereas staphylococci were reduced by 4.42 log levels. The blue wavelength of 450 nm was less effective, which is in accordance to the literature [46,47], reaching 4.92 log levels reduction for pseudomonads and 3.34 log levels for staphylococci at 1250 J/cm^2^. Noticeable is the distinct shoulder for irradiation of *P. fluorescens* with 450 nm, which does not seem to occur for *S. carnosus*, and is less pronounced at 405 nm irradiation.

### 2.2. Vitality Determination with ATP Assay

**Figure 2:**
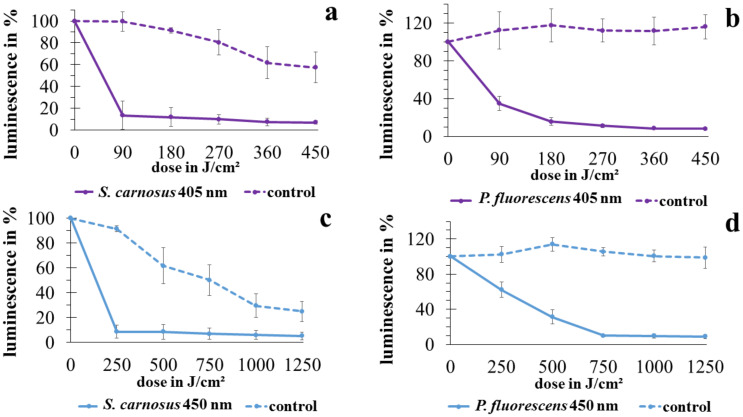
Luminescence progress, related to ATP content of *S. carnosus* irradiated with 405 nm (**a**) and 450 nm (**c**) as well as of *P. fluorescens* at 405 nm (**b**) and 450 nm (**d**). Error bars are representing the standard deviation of three independent experiments.

The results obtained with the ATP test are depicted in Figure 2. Different behavior for *P. fluorescens* and *S. carnosus* is obvious. The ATP level in the unirradiated control stays stable for *P. fluorescens* not only at the shorter 405 nm experiment (1 h 47 min), but also until 1250 J/cm^2^ of 450 nm, which is a 5 h duration with the applied intensity. The irradiated samples, however, experience a steep decrease already for the first sampling interval at 90 J/cm^2^ (21.5 min) and 250 J/cm^2^ (60 min) for the 405 nm and 450 nm irradiation, respectively. For *S. carnosus* not only the luminescence in the irradiated samples, but also the value in the non-irradiated control is considerably decreasing. Within 1 h 47 min (corresponding to 450 J/cm^2^ of 405 nm) the control sinks to 57.4%. For the longer blue light experiment of 5 h duration, 25.0% of the original fluorescence signal have been reached at the end of experiment in the control (corresponding to 1250 J/cm^2^ of 450 nm). The decrease in the control seems to follow a linear behavior. In contrary, the sample progression again shows a steep decrease in the first interval.

### 2.3. Fluorescence Staining

**Figure 3:**
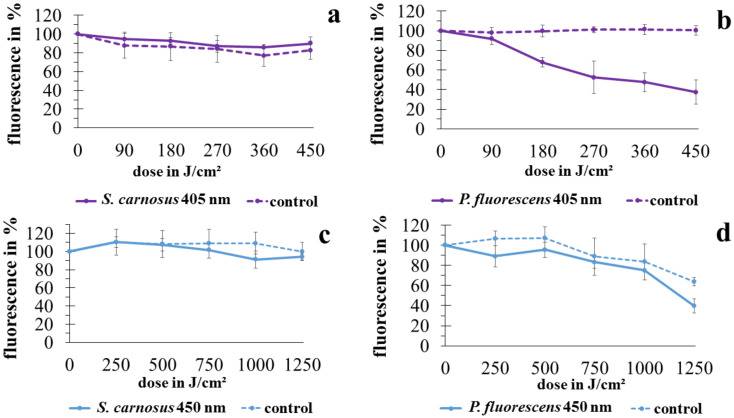
Fluorescence progress, related to membrane permeability for *S. carnosus* irradiated with 405 nm (**a**) and 450 nm (**c**) as well as for *P. fluorescens* at 405 nm (**b**) and 450 nm (**d**) irradiation. Error bars are representing the standard deviation of three independent experiments.

For investigation of the membrane integrity, the LIVE/DEAD^®^ BacLight™ kit was applied. In Figure 3, the relative fluorescence of SYTO9 referring to the start value is depicted. A decrease in fluorescence signal would demonstrate the entering of propidium iodide, indicating permeability of the membrane.

As already noticed in luminescence measurements, the two microorganisms *P. fluorescens* and *S. carnosus* react differently. For *S. carnosus* in both the control and the irradiated sample, no decrease in fluorescence signal could be noticed for 405 nm or 450 nm irradiation. For *P. fluorescens* at 405 nm irradiation the fluorescence signal in the control likewise stays stable. In the irradiated sample however, a pronounced decrease following a linear behavior to 37.4% at 450 J/cm^2^ irradiation is observable. In the 450 nm irradiation experiment, the control stays stable until 2 h (corresponding to 500 J/cm^2^ for irradiated sample) and then starts to decrease as well, reaching 63.8% at 1250 J/cm^2^. In contrary to the 405 nm experiment, at 450 nm irradiation there is only a slight fluorescence decrease in the sample to 75.2% at 1000 J/cm^2^ with a following steeper progression to 39.6% at 1250 J/cm^2^. Overall, the fluorescence signals for *P. fluorescens* at 450 nm irradiation proceed in parallel concerning control and sample. There does not seem to be a considerable effect of the irradiation on membrane integrity at this wavelength.

### 2.4. Transmission Electron Microscopy

**Figure 4:**
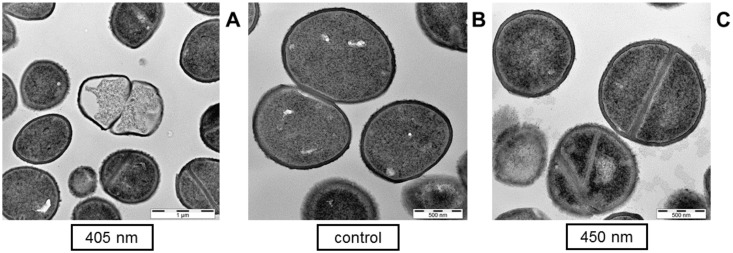
Transmission electron microscopy images of *S. carnosus* on single cell scale to assess bacterial cell integrity after visible light irradiation. Bacteria are depicted after irradiation with 90 J/cm^2^ of 405 nm (**A**), or irradiation with 400 J/cm^2^ of 450 nm (**C**) compared to the unirradiated control (**B**). Scale bars show the magnification, representing 1 µm (**A**) and 500 nm (**B**,**C**).

On the micrographs prepared in our study, individual bacterial cells revealed destructed appearance, and it is possible to contrast damaged irradiated samples with a healthy control on the single cell scale (Figure 4), similarly as it was conducted in the literature previously. Figure 4A demonstrates leakage of intracellular material, showing an empty cell envelope of *S. carnosus* after 90 J/cm^2^ of 405 nm irradiation. After 450 nm irradiation of 400 J/cm^2^ there was likewise the appearance of leaked cell material surrounding a cell with confused segmentation at cell division (Figure 4C).

**Figure 5:**
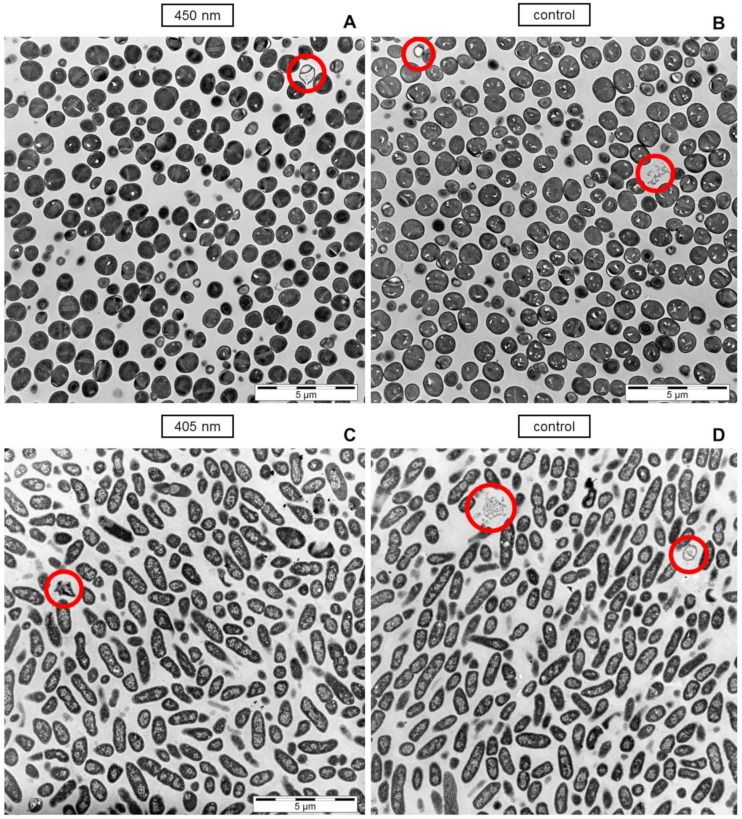
Transmission electron images of *S. carnosus* (**A**,**B**) and *P. fluorescens* (**C**,**D**) demonstrating on overview of bacterial appearance after 450 nm irradiation (**A**) and 405 nm irradiation (**C**) compared to the controls respectively (**B**,**D**). Circles are indicating potentially conspicuous individual cells. Scale bars show the magnification, representing 5 µm (**A**–**D**).

However, considering the overview at a lower magnification, no prominent bacterial lysis is observable in light treated cells compared to the controls, even though 90% of the cells should be damaged according to viable counts at the applied doses, respectively (Figure 5). Irradiation of 450 nm on *S. carnosus* demonstrated one emptied cell with leaked intracellular material at the top right corner of the image and some lighter shaded cells that might have lost intracellular material (Figure 5A), but the same occurs in the control (Figure 5B) demonstrating an emptied cell on the top left corner and some cell debris at centric right position. For *P. fluorescens,* single cell envelopes without intracellular material have only been visible on micrographs of the control (Figure 5D), while only moderately affected individual cells could be found on images of 405 nm irradiated samples. Images of *S. carnosus* irradiated with 405 nm and of *P. fluorescence* irradiated with 450 nm likewise show the same appearance as their respective controls (data not shown). 

According to viable colony counts 90% of microorganisms in the irradiated samples should appear compromised, but there is no considerable difference observable compared to the control on microscopic images.

## 3. Discussion

In this study, the potential damage of the membrane by visible light irradiation, which was often specified in the literature, was investigated. In our previous study on *S. cerevisiae*, damage of the membrane could not be detected [36]. As studies towards intracellular targets sometimes appear to contradict each other, we wanted to further elucidate membrane damage, examining *S. carnosus* and *P. fluorescens* as examples for a Gram positive and a Gram negative bacterial species. While those species are non-pathogenic, great differences between pathogenic and non-pathogenic species are not expectable concerning the incurred damage, due to non-specificity of the photoinactivation mechanism by means of ROS. Indeed, it was demonstrated before [48], that the same strains, which have also been applied here, reacted similarly (405 nm) or even less susceptible (450 nm) towards photoinactivation than their pathogenic relatives. Since we made sure to reach an overall inactivation of over three log levels for both microorganisms and wavelengths, respectively, potential damages occurring for bacteria exposed to visible light should be observable within this investigation.

A lack of stained cells, following trypan blue treatment to evaluate membrane damage after irradiation with 405 nm and 450 nm, was already observed in *S. cerevisiae* [36], whereas a considerable reduction of colony counts was noted. The reduction in CFU of *S. cerevisiae* in combination with unstained cells could rather point to intact membranes due to other impairment mechanisms, or to undamaged cells that solely lost culturability. Therefore, in the current study, we added an ATP test, to make sure that the findings are not based on a sole reduction in culturability. The decrease in ATP level, which we observed for *S. carnosus* and *P. fluorescens*, indeed showed an impact of visible light irradiation on bacterial vitality. While the control stayed stable in *P. fluorescens*, the luminescence indicating the ATP level sank rapidly in irradiated samples for both 405 nm and 450 nm.

For *S. carnosus,* the luminescence decreased over time already in the control. After 1 h 47 min (related to 450 J/cm^2^ at 405 nm), only 57.4% ATP level was reached sinking further to 25.0% after 5 h (related to 1250 J/cm^2^ at 450 nm). However, the CFU of the control did not decrease. The energy consumption over time during storage in phosphate buffered saline (PBS) seems to be higher for *S. carnosus* than for *P. fluorescens*. In irradiated samples however, the luminescence already distinctly decreased within the first interval with a clear difference to the control, probably due to the loss of vitality by visible light. Sassoubre et al. [49] investigated enterococci under sunlight influence in seawater concerning photostress, applying an ATP test among diverse methods under light, dark, oxic, and anoxic conditions. For all light conditions, ATP decreased steeper than for the control in the dark, but still there existed decrease in the control, similar as in the current study. Remaining ATP in the irradiated sample at the end of experiment was explained by already nonculturable cells being in a transient state between viability and death, still harboring ATP.

A quick ATP depletion compared to other viability indicators was also observed in several studies irradiating with UV-A light (315–380 nm) or pulsed broad band light [50,51,52], with the conclusion that cells might die from ATP exhaustion and the following loss of membrane potential [51]. In the study of Berney et al. [50], for example, the reduction in glucose uptake correlated well with the loss of membrane potential and at the same time with culturability. Quick ATP decrease, as also observed in the current study, might be caused by the attempt of maintaining membrane potential or other recovery processes [51]. A certain level of ATP was nevertheless still maintained even after high doses and after other indicators demonstrated sequential loss of cellular functions [50], similar as noticed in the current study. The mechanism for UV-A inactivation might differ from visible light irradiation. However, also for 405 nm irradiation deprived membrane functionality was demonstrated by flow cytometry, demonstrating non-pumping cells [21]. Loss of phosphate and potassium was shown on *Propionibacterium acnes*, ascribed to dysfunctional ion pumps [4]. Likewise, the immediate change in membrane potential within 5 min shown by Biener et al. [17] points to inactivation of ion pumps. Inactivation of ion pumps was also concluded from observations concerning transmembrane potential and K^+^ leakage in *Staphylococcus aureus* [31]. The impairment of pumps that have been observed in the mentioned studies might have its cause in the similarly quick depletion of ATP that was demonstrated in the current study. This points to a loss of membrane functionality rather than the direct destruction of membrane structures, as it was suggested for photoinactivation before.

Multiple studies towards the mechanisms of visible light photoinactivation at violet (400–420 nm) and blue (450–470 nm) light have been conducted revealing different possibilities of cellular damages. For violet visible light irradiation no lipid peroxidation could be found in *Salmonella enterica* [22], nor in *Porphyromonas gingivalis* [53] after colony reducing doses and only in small amounts in *Cronobacter sakazakii* [54], but was considerable for *S. aureus* after a sub-lethal dose [31]. These results substantiate our assumption that direct membrane destruction might not be a substantial cause of microbial death. Furthermore, no permeabilization of the membrane was observed for *S. cerevisiae* [23] and *S. enterica* [21]. A decrease of membrane integrity as a function of photosensitizer concentration was noticed in antimicrobial photodynamic inactivation (aPDI) applying external photosensitizers [55], but high concentrations of photosensitizers are necessary to destroy the membrane. According to Kim et al. [22], the endogenous produced amount of photosensitizers could be too small to act oxidative on lipids. Several authors already published electron microscopy images after visible light irradiation for violet [4,16,21,27,28,30,56] and blue wavelengths [29]. Mainly intracellular damage and/or formation of vacuoles was observed in *Pseudomonas aeruginosa*, *S. aureus*, *Acinetobacter baumannii*, *Candida albicans*, *P. acnes*, *S. enterica,* and *Escherichia* coli [4,21,27,28,30,56]. However from the appearance of totally destroyed cells of *S. aureus* [16], significant leakage of intracellular substance in *A. baumannii* [27] as well as disrupted cell walls, and complete loss of cytoplasmic components at higher doses in *C. albicans [28]*, and release of intracellular components of *Listeria monocytogenes* and *P. fluorescens* after 4 days of exposure to visible light [29], it was concluded that membrane permeability must play an important role in photoinactivation processes. Scanning electron microscopy images depicted structures that looked like holes on *Helicobacter pylori* after 9.3 J/cm^2^ [57]. 

Besides the mentioned impairments that have been detected, several other studies confirmed nucleic acids being attacked by visible violet [20,21,22] and blue light [24,53,58], as well as genetic responses in oxidative stress pathways [25,26]. Furthermore, sensitivity to high salt and bile media was increased in *S. aureus*, *E. coli*, *Bacillus cereus*, L. *monocytogenes*, *S. enterica*, and *Shigella sonnei* by violet light irradiation [15,18,19].

Even if there are no Gram dependent differences in overall impact of photoinactivation based on endogenous photosensitizers [46,47], there are still different behaviors recognizable. The results in this study demonstrate different reactions concerning fluorescence staining between Gram positive *S. carnosus* and Gram negative *P. fluorescens*. For the Gram negative species significant uptake of propidium iodide (PI) occurred at 405 nm irradiation while for the Gram positive species nothing similar was observed. The results of McKenzie et al. [15] are in total accordance to our results concerning Gram differences. The structural differences might play a role for this observation. Nevertheless, more species have to be tested concerning Gram differences with a demonstration of varying behavior, before drawing a general conclusion about a causal relation. At 450 nm irradiation on *P. fluorescens*, though there was a decrease in fluorescence in the sample, the control sank similarly, with the difference between sample and control not being pronounced.

An explanation for PI entering, independent of membrane destruction, might be given by the membrane depolarization caused by loss of membrane functions. Loss of potassium was already noticed before for 405 nm light irradiation in *P. acnes* [4] and inactivation of ion pumps was also hypothesized for *S. aureus* [17,31].

Kirchhoff and Cypionka [59] artificially produced an altered membrane potential in Gram negative *Dinoroseobacter shibae* by anoxic conditions. The latter induced a high membrane potential (negative intracellular), due to loss of potassium, which led to appearance of red stained cells. However, this effect was reversible within 5 min when the culture was allowed to recover in oxic conditions, with the cells appearing green again. Additional membrane potential staining with 3,3′-Diethyloxacarbocyanine Iodide (DiOC_2_(3)), demonstrated the recovery to normal membrane potential in correlation with the decline of red fluorescence staining. As PI carries two positive charges the ion motive force for bacteria, highly negative intracellular, can lead to a breakthrough of the stain without permeabilized membrane. The authors concluded that the results of live/dead staining can be biased by the membrane potential of the stained cells independent of membrane permeability.

The presence of strongly negatively charged lipopolysaccharides in the outer membrane of Gram negative bacteria might lead to increased binding affinity of the positively charged PI to Gram negative species and therefore facilitate this process effect. Furthermore, irradiation with violet light of 405 nm seems to enhance the phenomenon compared to the control.

However, Kim et al. [21] only found a membrane depolarization of less than 10% in spite of the indicated loss of membrane functionality due to extensive inactivation of both the efflux pumps and the glucose uptake system. Another possibility of PI uptake independent of depolarization therefore could be the destruction of membrane proteins by ROS. Membrane proteins often play a role in transport, such as Na^+^K^+^ pump, which was suggested to be impaired by Wu et al. [31] measuring increased K^+^ leakage after irradiation. This would also explain the sole occurrence of PI uptake in Gram negative *P. fluorescens* in this study and the similar results from McKenzie et al. [15], as Gram negative species possess a greater number of such membrane bound proteins. McKenzie et al. [15] demonstrated uptake of SYTOX green, which carries three positive charges [60], also only for the investigated Gram negative microorganism irradiated with 405 nm.

In our results additionally moderate uptake of PI occurred after prolonged storage in PBS in the control, to the same extent as for 450 nm irradiation. It is therefore unclear whether 450 nm irradiation provokes PI uptake in *P. fluorescens*. The difference between the two wavelengths might be due to different photosensitizers responsible for induction of ROS formation with porphyrins at 405 nm and flavins at 450 nm [8,9,10,11,12,61]. 

Occurrence of PI stained bacteria for Gram positive species, in contrary to our study and the one of McKenzie et al. [15], have been found in the literature for blue light irradiation [26,29] as well as for violet light [18]. However, Hyun and Lee [29] performed their observations after a long irradiation time of 4 days, which might have led to PI uptake even in Gram positive representatives. Kim et al. [18] detected micrographs with red cells for Gram positive *B. cereus*, *L. monocytogenes* and *S. aureus* under refrigeration temperature, which might have further influenced intracellular actions. For observations from Yang et al. [26], it stays unclear whether they were performed in medium instead of PBS, which would shift experiments towards aPDI processes due to potential external photoactive compounds. Equally PI staining was observed for the yeast *C. albicans* determined with a single stain technique of high PI concentration [62].

The majority of studies demonstrated intracellular damages through visible light irradiation [4,21,27,28,30,56]. Still, completely destroyed bacteria and leakage of intracellular components was observed by measurement of absorbance at 260 nm and on TEM images [15,16,27,28,29,30]. The degree of destruction in those cases was extremely high. Instead of small holes in the membrane, the cells rather showed a shredded impression. Those damages could be of a kind that are not ROS induced, but due to disintegration of dead cells, which were inactivated by other causes than membrane damage. Dynamically investigated *S. cerevisiae* under 415 nm irradiation [23] demonstrated a sudden expulsion of intracellular components, while the membrane itself was intact until this event. Increased absorbance at 260 nm in the supernatant [15] could also be explained out of disintegrated cell envelopes, occurring just after cell death instead of being a part of the cause of death by photoinactivation. On the TEM images performed in this study, we could only detect individual cells with destroyed appearance, but the entirety of the sample did not show any difference to the control, demonstrating intact cell envelopes and barely destructed cells. Wu et al. [31] came to similar conclusions investigating 415 nm irradiated *S. aureus* with atomic force microscopy finding intact cell morphologies.

An interpretation of the mechanism towards mostly intact lipid membranes combined with the possibility of PI uptake, especially in Gram negative strains, due to membrane depolarization, would explain our results on *S. cerevisiae* [36]. The lack of trypan blue stained cells after irradiation might be based on trypan blue’s negative charge, compared to the positive charge of PI. At the same time, most results of other literature studies match the assumption of intact cell envelopes but lost membrane functionality. It is in accordance to the lack of lipid peroxidation evidence in some studies [22,53] and to a study of Kim and Yuk [21] coming to similar conclusions. The authors express a related assumption from their results, including the effect mainly being attributed to DNA oxidation and a loss of membrane functions, such as efflux pumps and glucose uptake system, rather than membrane disruption. Membrane depolarization due to potassium and phosphate loss was noticed before [4] and ATP depletion was mentioned as possible cause. The inactivation of ion pumps and the following alteration of vital cellular functions has been hypothesized out of increased transmembrane potential measurements [17] and K^+^ leakage [31]. Disrupted appearance on TEM images and leakage of intracellular material, noticed in the literature, is suggested to be found for disintegrating bacteria that have been inactivated due to other impairments than membrane destruction. Increased sensitivity towards high salt and bile concentrations [15,18,19] appears logical, taking into account a depolarized membrane due to membrane functionality loss. Already in UV-A irradiation studies similar conclusions have been drawn. The first level of damage was described to be the change of membrane potential going along with loss of culturability. Only after prolonged irradiation and after culturability was already below detection limit, the membrane integrity was lost [50].

## 4. Materials and Methods

### 4.1. Bacterial Strains

Bacteria investigated in this study were obtained from DSMZ (Deutsche Sammlung für Mikroorganismen und Zellkulturen, Braunschweig, Germany). Due to safety regulations in the available laboratory, it is not possible to cultivate pathogenic strains there. However, it was previously demonstrated that non-pathogenic representatives reacted similar or even less susceptible towards visible light photoinactivation as their pathogenic relatives [48]. *Pseudomonas fluorescens* (DSM4358) were cultivated in 535 medium (30 g tryptic soy broth (Sigma-Aldrich Chemie GmbH, München, Germany) per liter) at 30 °C and *Staphylococcus carnosus* (DSM20501) were cultivated in M92 medium (30 g tryptic soy broth (Sigma-Aldrich Chemie GmbH, München, Germany), 3 g yeast extract (Merck KGaA, Darmstadt, Germany) per liter) at 37 °C at rotary conditions of 170 rpm from an overnight preculture in 30 mL fresh medium until mid-exponential phase (optical density at 600 nm OD_600_ = 0.35) was reached. Bacterial cultures were centrifuged at 7000× *g* for 5 min and the resultant pellet resuspended in PBS, washed in PBS and diluted to 1–5 × 10^7^ CFU/mL for experimental use.

### 4.2. Irradiation Setup

Two different wavelengths were applied in this investigation with LEDs (light emitting diodes) as light source. Violet light at 405 nm (LZ4-40UB00-00U8 (LED Engin, Inc., San Jose, CA, USA.) and blue light at 450 nm (LZ4-00B208 (LED Engin, Inc., San Jose, CA, USA.) were applied, as those are the peak wavelengths of porphyrin and flavin absorption, supposed to be the responsible photosensitizers for bacterial light inactivation. The emission spectra of the LEDs were measured with a spectrometer (SensLine AvaSpec-2048 XL, Avantes, Appelsdorn, The Netherlands) at its operating current after a pre-heating interval. The LEDs were mounted to a heat sink, additionally cooled with an active fan, placed on top of a truncated hollow pyramid with a high reflective inside (described before in [10,48]). This procedure ensured homogenous irradiation intensity within the sample area. An irradiation intensity of 70 mW/cm^2^ was adjusted within this setup using an optical power meter OPM150 (Qioptiq, Göttingen, Germany) and checked at every sample drawing interval. 3 mL of the bacterial suspension were filled in a 5 mL beaker glass and positioned in a black bracket designed for holding the beaker glass, which was set to a small water bath with black walls and bottom. To avoid rise of temperature within the sample during irradiation, the water bath was cooled, with the temperature set at 20 °C, which was verified contactless at every sample drawing interval by means of an infrared thermometer Raynger MX (Raytek Fluke Process Instruments GmbH, Berlin, Germany). The black surrounding ensured that only light from above, as measured with the power meter, would reach the sample and results would not be falsified by reflections from the white laboratory tables.

### 4.3. Viability Determination with Colony Forming Units

Samples were drawn after homogenizing the solution every 21.5 min for 405 nm and every 60 min for 450 nm irradiation, resulting in intervals of 90 J/cm^2^ and 250 J/cm^2^, respectively. After serial dilution in PBS to proper densities, 33 µL sample volumes were plated on agar with glass spatula. Plates were enumerated manually, after incubation for 24 h at 37 °C for *S. carnosus* and 48 h incubation at 30 °C for *P. fluorescens.* The resultant counts were converted in log levels of reduction. Each sample was tested in triplicates and each experiment was repeated three times.

### 4.4. Vitality Determination with ATP Assay

To investigate the vitality of irradiated bacteria ATP was chosen as indicator, tested with the BacTiter-Glo™ Microbial Cell Viability Assay (Promega, Madison, WI, USA). This assay allows to directly add a single reagent to the culture for measuring resulting luminescence, avoiding pipetting errors. As bacteria are lysed, intracellular ATP level can be determined by the luminescent signal, which is proportional to the ATP level. BacTiter-Glo™ Reagent was put in aliquots stored at −70 °C and was equilibrated to room temperature before use. 50 µL of reagent were added to 50 µL of irradiated bacterial sample inside a white 96-well plate. The plate was immediately transferred to the microplate reader CLARIOstar Plus (BMG Labtech, Ortenberg, Germany) at 30 °C, where samples were orbitally shaken and then incubated for 5 min prior to luminescence recording. The temperature, slightly above room temperature, was chosen to ensure equal conditions for all samples independent of room temperature rise in summer months. All samples were tested in triplicates. Samples of non-irradiated controls were equally prepared and tested in triplicates at each irradiation interval. All experiments were repeated three times. At the beginning of each independent experiment, an ATP-standard was measured to guarantee the functionality of the reagent.

### 4.5. Fluorescence Staining 

We applied fluorescence staining of irradiated bacterial samples to investigate potential loss of membrane integrity. The LIVE/DEAD^®^ BacLight™ kit (Thermo Fischer Scientific, Waltham, MA, USA), which is in common use for determining live/dead ratios, was applied for membrane integrity tests. It is based on two fluorescent stains of which SYTO9 generally enters all cells, whereas propidium iodide (PI) can only penetrate membranes with existing damage. The green fluorescence of SYTO9 is reduced in the presence of the red fluorescent PI; hence, making it possible to distinguish between live and dead cells, provided that inactivation accompanies membrane defects [18,50,63]. SYTO9 dye and propidium iodide (PI) from the LIVE/DEAD^®^ BacLight™ Bacterial Viability kit were stored at −20 °C and mixed in equal volumes directly before use. To a 100 µL sample 0.2 µL dye mixture was added and incubated at room temperature in the dark for 15 min. Fluorescence measurements were performed in a black 96-well plate with the microplate reader CLARIOstar Plus (BMG Labtech, Ortenberg, Germany) at 30 °C after orbital shaking. Excitation wavelength for the spectrum scan was set to 485 nm ± 5 nm at a gain of 1750 and the scan was performed between 510 nm and 740 nm. The excitation wavelength of 485 nm allowed to simultaneously excite both fluorescent dyes in one test run, as SYTO9 had its excitation maximum at 480 nm and PI at 490 nm. Emission was detected at 530 nm for SYTO9 and 630 nm for PI by integrating the fluorescent scan within a range of ±2 nm around those assigned wavelengths. The fluorescence signal emitted by PI around 630 nm will not change much, while the penetration of PI inside the cell is indicated by attenuation of the fluorescence signal of SYTO9 around 530 nm [64]. A reference curve was established by heat killing bacteria at 95 °C for 5 min and mixing those samples with the original suspension in proportions of 0%, 25%, 50%, 75%, and 100% to reach clear conditions of live and dead microorganisms. Each sample was tested in duplicates and each experiment was repeated three times.

### 4.6. Transmission Electron Microscopy

In a 48 well microtiter plate, 15 wells with a volume of 1 mL each of the working suspension with 1–5 × 10^7^ CFU/mL bacterial concentration were irradiated simultaneously and homogenized afterwards to attain a sufficient number of bacteria for microscopic analysis. For the 405 nm irradiation at 70 mW/cm^2^ a dose of 90 J/cm^2^ was chosen for both *S. carnosus* and *P. fluorescens* to achieve a 90% inactivation according to colony counts. For 450 nm a dose of 500 J/cm^2^ was chosen for *P. fluorescens* and 400 J/cm^2^ for *S. carnosus* with the objective to see mainly inactivated bacteria on the micrographs. After centrifugation for 3 min at 7000× *g* the supernatant was discarded and the resulting pellet with a residual liquid of 20 µL was resuspended in 20 µL of a double concentrated fixative containing 5% glutaraldehyde and 2% sucrose in phosphate buffer (pH 7.3). After 5 washing cycles with phosphate buffer, the samples were postfixed in 2% aqueous osmium tetroxide followed by dehydration in a graded series of 1-propanol. Samples were blockstained in 1% uranyl acetate and embedded in Epon. Ultra-thin sections (80 nm) were collected on copper grids and contrasted with 0.3% lead citrate for 1 min. The final samples were imaged in a JEOL TEM 1400.

## 5. Conclusions

Our investigation on Gram negative *P. fluorescens* and Gram positive *S. carnosus* showed the reduction of colony forming units by more than three log levels with both 405 nm and 450 nm, with higher doses required for the blue wavelength. This colony reduction is probably caused by inactivation instead of sole loss of culturability, as the tested ATP level decreased. However, a certain level of ATP remained even at high doses and low CFU. ATP might be stored intracellular within intact cell envelopes independent of a viable or death state of the microorganism. Propidium iodide uptake was only observed in Gram negative *P. fluorescens,* but not in Gram positive *S. carnosus*. However, we ascribe the PI uptake to a loss of membrane functionality without its direct damage. This might be due to the increase of membrane depolarization or to inactivation of membrane proteins. TEM images did not indicate considerable disruption of cell envelopes as appearance was similar to the control. We conclude from these pictures that microscopically easy identifiable cell disruption is not a key factor for the inactivation with visible light. The role of the membrane in photoinactivation with endogenous photosensitizers might be more directed concerning functional impairment rather than destructive permeabilization. However, further investigation is needed concerning the plausibility of this theory.

## Data Availability

The data presented in this study are available on request from the corresponding author.
